# Personality and Social Framing in Privacy Decision-Making: A Study on Cookie Acceptance

**DOI:** 10.3389/fpsyg.2016.01341

**Published:** 2016-09-07

**Authors:** Lynne M. Coventry, Debora Jeske, John M. Blythe, James Turland, Pam Briggs

**Affiliations:** ^1^Psychology and Communication Technology Lab, Department of Psychology, Northumbria UniversityNewcastle upon Tyne, UK; ^2^Business School, Edinburgh Napier UniversityEdinburgh, UK; ^3^School of Computing Science, Newcastle UniversityNewcastle upon Tyne, UK

**Keywords:** privacy, cookie, nudge, risk-taking, impulsivity, social norms

## Abstract

Despite their best intentions, people struggle with the realities of privacy protection and will often sacrifice privacy for convenience in their online activities. Individuals show systematic, personality dependent differences in their privacy decision making, which makes it interesting for those who seek to design ‘nudges’ designed to manipulate privacy behaviors. We explore such effects in a cookie decision task. Two hundred and ninety participants were given an incidental website review task that masked the true aim of the study. At the task outset, they were asked whether they wanted to accept a cookie in a message that either contained a social framing ‘nudge’ (they were told that either a majority or a minority of users like themselves had accepted the cookie) or contained no information about social norms (control). At the end of the task, participants were asked to complete a range of personality assessments (impulsivity, risk-taking, willingness to self-disclose and sociability). We found social framing to be an effective behavioral nudge, reducing cookie acceptance in the minority social norm condition. Further, we found personality effects in that those scoring highly on risk-taking and impulsivity were significantly more likely to accept the cookie. Finally, we found that the application of a social nudge could attenuate the personality effects of impulsivity and risk-taking. We explore the implications for those working in the privacy-by-design space.

## Introduction

People value their privacy, but do not readily protect it – a phenomenon known as the privacy paradox (Smith et al., 2011). There are a variety of explanations for this: people may not be aware of the various ways in which they leave themselves vulnerable online; they may not know how to protect themselves; they may find the act of establishing and maintaining privacy protection online too onerous or they may simply be willing to sacrifice their privacy in some kind of implicit or explicit trade – for convenience, for goods or simply for a better, personalized service.

Privacy is a fundamental issue for those involved in human–computer interaction. Human–computer interaction researchers may seek to build privacy considerations into their work from the outset – adopting a *privacy by design* approach (Brown, 2014) that embeds privacy thinking into the entire R&D lifecycle, ensuring that privacy is a core component in the final design. Or they may consider privacy ‘bolt-ons’ to final systems in the form of new functionality, new forcing functions or choice architectures that attempt to ‘nudge’ users to behave in ways that offer greater privacy protection. The latter is often discussed in terms of the ‘economics of privacy’ (e.g., Acquisti et al., 2013), recognizing that users’ privacy decisions are not necessarily stable over time, but rather reflect the economic and social costs and benefits of protecting privacy within a particular context. The wider context for privacy decision-making may include those personality variables that directly affect an individual’s ability to tolerate task interruptions as well as a range of task framing effects that make privacy risks more or less salient. In other words: “*what people decide their data is worth depends critically on the context in which they are asked - specifically, how the problem is framed*” (Acquisti et al., 2013, p.32).

This paper explores some of the framing and personality influencers that affect privacy-related decision making, specifically in the context of accepting or rejecting cookies – a known issue in relation to privacy. Participants were presented with an online travel shopping task and asked to judge trustworthiness, familiarity and likelihood of using of four different sites with the intention of masking the true aim of the study, i.e., cookie acceptance. Within this task the acceptance or rejection of cookies was framed by presenting population norms for this specific behavior (a social framing manipulation). The paper also considers the influence of different personality traits on responses to these framing manipulations. Nudges presently give little attention to personality differences assuming that all individuals are homogenous. The idea is that the presentation of choices can be made in such a way as to ‘nudge’ people toward a particular decision (Thaler and Sunstein, 2008), and that these nudges can cater for different personality traits that will be more or less susceptible to such nudges. Nudges that use social norm references are of particular interest as they can influence individuals to mimic the behaviors and decisions of a group by appealing to the need for group affiliation and social conformity.

Social norms have been used to influence behavior across a variety of contexts including health decision-making (Jetten et al., 2012); energy conservation (e.g., Allcott, 2011) and tax compliance (Posner, 2000). For example, using descriptive social norms (statements about how other individuals have acted in the given situation), researchers have compared numerical majority norms (e.g., 73 or 74%) to minority norms (e.g., 27 or 37%) and found that these simple manipulations can increase fruit consumption (e.g., Stok et al., 2012) or increase voting participation amongst students (Glynn et al., 2009). These studies have effectively demonstrated that individuals will use information about others like themselves to help make decisions. Majority norms are believed to be particularly effective as they refer to how most people behave in a certain situation and provide consensus information to individuals over what the ‘correct’ behavior is perceived to be (Thibaut and Kelley, 1959). However, a study by Besmer et al. (2010) suggests that minority norms may be more effective in influencing privacy decisions.

Social norms have also been used to manipulate the adoption of available privacy or security solutions (Das et al., 2014), although we should note that results here have been mixed. Social framing has been used to improve cookie management (Goecks and Mynatt, 2005), the creation and maintenance of personal firewalls (Goecks et al., 2009) and for peer-to-peer file sharing (DiGioia and Dourish, 2005). However, they are not always associated with the strongest effects on behavior change. For example, Patil et al. (2011) explored social influences on the privacy settings on Instant Messenger, finding that social cues were influential as a secondary manipulation, but showed very small effect sizes in comparison to other privacy manipulations. Further, we should be mindful that social nudges can work both ways – i.e., they can lead to a greater willingness to divulge sensitive information when informed that others have made similar disclosures (Acquisti et al., 2012). Further work is required in the privacy domain to resolve the role and effectiveness of social norm framing for privacy decisions in order to clarify the circumstances in which social framing might have limited or no influence (Anderson and Agarwal, 2010; Patil et al., 2011; Knijnenburg and Kobsa, 2013) or the conditions under which minority vs. majority social norms are effective (Besmer et al., 2010). One particularly neglected condition is the role of personality in decision-making.

Decisions can be mapped to personality in a number of ways (Riquelme and Román, 2014). For example, collaborative decision-making can reflect the extent to which individual’s exhibit social or individual value orientations (e.g., Steinel and De Dreu, 2004) or might reflect their collaborative vs. competitive tendencies (McClintock and Liebrand, 1988). Risky decisions are more likely to be made by those individuals who score high on openness to experience, while those who score highly on neuroticism are more likely to be risk-averse, at least in the domain of gains (Lauriola and Levin, 2001). Existing research also suggests that personality factors may interact with social manipulations to influence decision-making as personality traits may often affect behavior indirectly through influencing normative determinants of behavior (Rosenstock, 1974; Ajzen, 1991). In the security and privacy domains, a range of candidate personality traits can be identified that are likely to affect decision-making, including impulsivity, risk-taking/risk propensity (Grossklags and Reitter, 2014), willingness to self-disclose information about oneself, and sociability. These are described in more detail below.

Impulsivity, or impulsiveness, describes the tendency of individuals to give insufficient attention to the consequences of an action before carrying it out or being aware of the risk involved. Impulsivity may lead individuals to make decisions without having all the facts when processing information (Eysenck et al., 1985; Dickman, 1990; Franken and Muris, 2005). This lack of premeditation and deliberation (Halpern, 2002; Magid and Colder, 2007) as well as greater distractibility (Stanford et al., 2009) may then result in decisions that not only lead to regret, but also increase the potential vulnerability to risk for the more impulsive individual. Research indicates impulsivity may be thwarted under negative normative evaluations and heightened under positive normative evaluations (Rook and Fisher, 1995). Higher impulsivity is associated with lower self-control (Romer et al., 2010; Chen and Vazsonyi, 2011). When self-control resources are low, people are more likely to conform to social persuasion (Wills et al., 2010; Jacobson et al., 2011), especially when self-control is depleted and resistance has broken down (Burkley et al., 2011). In the absence of self-control to fend off persuasive appeals, people may become more susceptible to social influence.

Risk-taking is another interesting trait. This trait is influenced by past experience but also typically changes with age (Mata et al., 2011) and gender (Rolison et al., 2012). While impulsive people act on the spur of the moment without being aware of the risks involved, risk takers are aware of the risk and are prepared to take the chance (Dahlbäck, 1990). Risk-takers are less concerned about the repercussions of their actions as compared to risk-averse individuals who are more likely to be concerned about subsequent regret (Zeelenberg et al., 1996). In addition, risk-takers are more likely to make decisions ‘in the moment’ and to be influenced by emotional cues or mood (e.g., boredom, stress, disengagement) rather than a rational calculation (Figner and Weber, 2011; Visschers et al., 2012). Perhaps unsurprisingly, risk takers have also been shown to be less concerned about privacy (Egelman and Peer, 2015).

The third trait of interest is willingness to self-disclose, reflecting the fact that individuals vary in their willingness to share information about the self. Evidence for this comes from social networking research (Kaplan and Haenlein, 2010), health research (Esere et al., 2012) and also consumer research (Moon, 2010). A greater willingness to self-disclose information may render individuals vulnerable to privacy and security risks, as they may not think through the consequences of this exposure (Acquisti and Gross, 2006). Individuals are more willing to disclose information when under social influence (Sánchez et al., 2014) and pay less attention to potential privacy risks when under this influence (Cheung et al., 2015).

The fourth and final trait of interest is sociability – a facet of extraversion (McCrae and Costa, 1985) and a trait that correlates positively with consumer trust in online retailers (Riquelme and Román, 2014). Sociability may be associated with a greater interest in relationship management and information sharing, motives that have also been examined in relation to voluntary self-disclosure (Lee et al., 2008). Extraverts are particularly sensitive to social attention (Lucas et al., 2000) but less likely to yield to social pressure (Sharma and Malhotra, 2007) and slower to learn social norms (Eysenck, 1990).

Finally, social norm information has been shown to moderate the relationship between personality and behavior. Rook and Fisher (1995) showed that social normative evaluations influenced consumers’ impulsive buying behavior. Specifically, they only found a significant relationship between impulsivity and buying behaviors if consumers believe that acting on impulse is appropriate. With regards to social norms and risk taking, social norms are often communicated to those exhibiting risky health behavior in order to reduce such behavior (e.g., Scholly et al., 2005; Baumgartner et al., 2010). Showing such vulnerable individuals that they are in the minority helps convince them that the behavior is inappropriate.

Cookie acceptance is a form of privacy behavior that has been relatively understudied in existing research and represents an opportunity to explore the effects of social framing and the potential interacting role of personality on an objective behavior. A cookie is a small piece of data sent from a website, which is then stored on a user’s browser and transmitted back to the website every time the user browses a site. Cookies are promoted as being necessary to enhance the user’s experience by aiding navigation, identifying preferences, allowing personalization, targeted advertising and remembering login credentials. However, cookies also raise a number of privacy and anonymity issues as they track user behavior and collect user-specific information within and across websites (see BBC, 2011; Opentracker, 2014). Cookies cannot contain viruses or malware, but they can be associated with a number of security issues as they can hold information such as passwords used in authentication and also the credit card details, addresses and similar to support automatic completion of online forms. The security of the information contained within the cookie is dependent on the security of the website, the security of the user’s web browser and computer and whether or not the data is strongly encrypted.

In 2002, an EU directive was introduced which required online providers to seek consent to the use of cookies and in 2011 it became law. This initially meant that users had to give their explicit consent for cookie use before continuing with any web-based interaction and European users quickly became familiar with a cookie dialog in which they would accept or reject cookies as an integral component in online exchange. The Commission went so far as to develop a ‘Cookie Consent Kit’ with the necessary JavaScript to enable the easy adoption of a cookie dialog on any website (Information Provider’s Guide, 2015). Subsequent legislation gave limited exemption to certain forms of interaction. Amid criticisms about how users are tracked and systems extract personal data (see review by Peacock, 2014), data protection concerns have further increased and new EU legislation on data protection (the General Data Protection Regulation) will be coming into force in 2018 as part of the European data privacy framework. These guidelines are also expected to lead to improved privacy protection for users via a new drive for the more widespread employment of a *privacy by design* philosophy. The framework will also make privacy impact assessments mandatory, while setting the stage for fines for those companies that breach the Great Britain (1998). This also means that they are responsible for the safe-keeping of data they obtain from various sources (Hawthorn, 2015) and the securing of appropriate consent. The information collected via cookies is likely to fall under this new remit.

At present, explicit cookie acceptance typically involves a dialog that requires the user to click an “accept” or “proceed” button to get access to a website. This is a typical dialog structure, similar to others that might signal the acceptance of terms and conditions, granting permission for a software download, or agreeing to mobile applications accessing information from a mobile device. We know that users habituate to such messages over time (i.e., the more frequently they see the message, the less attention they give them Böhme and Köpsell, 2010; Raja et al., 2010). Further, we know that dialog boxes often interrupt the user in achieving his or her primary goal. This habituation to dialogs coupled with the users’ relentless focus on the primary task can create security vulnerabilities as, for example, when users automatically accept cookies or select an ‘update later’ button that will enable them to continue with the job at hand (Zhang-Kennedy et al., 2014). It is useful, then, to carefully consider cookie dialogs more carefully both in terms of the design of the dialog itself as well as a consideration of any personality dimensions that might weaken privacy decision-making (such as impulsivity in accepting everything that looks like a cookie). The current study attempts to address these issues.

In this paper the following research questions are explored. First, to what extent can social framing manipulations influence privacy decision-making, expressed here in terms of cookie acceptance? Second, are there personality factors that determine how users are likely to respond in a privacy context and third, might these lead to differential responses to social nudges? Associated hypotheses are as follows.

## Social Framing

In comparison to a base rate (control) which is likely to lean toward acceptance of cookies, participants’ responses will be affected by a social nudge, such that a low social norm (minority) reference will effectively nudge participants away from accepting cookies.

### Personality

Cookies are more likely to be accepted when individuals are more impulsive and willing to share information, when they are greater risk takers and more sociable.

### Personality and Social Framing

Differences in cookie acceptance attributed to personality may be qualified by the effects of the social framing manipulation.

## Materials and Methods

### Design

The study design involved an incidental task in which participants were asked to review a series of travel websites and make a series of judgments about them. Before clicking on the sites to review, participants were presented with one of three cookie dialogs, representing three social framing conditions (control, minority social norm, majority social norm). The dependent measure for the true task was cookie acceptance.

**FIGURE 1:**
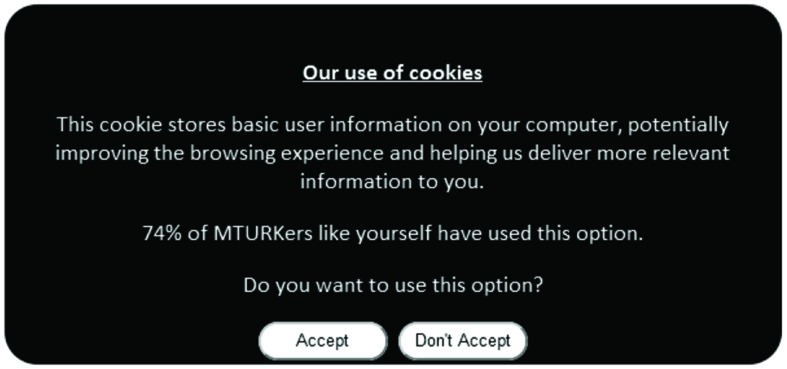
**Example of cookie dialog (displaying the majority social norm message)**.

The social framing manipulations were constructed as follows. The original (control) text in the cookie dialog box was similar to that recommended by the EU cookie directive and read as follows: “Our use of cookies. This cookie stores basic user information on your computer, potentially improving the browsing experience and helping us deliver more relevant information to you.” Two experimental conditions were then generated with additional text that made reference to either a minority or a majority social norm, as follows: “37% (74%) of MTURKers like yourself have used this option.” All cookie dialogs concluded with: “Do you want to use this option? Accept/Don’t accept” (see **Figure 1**). Allocation to each of the three conditions (control, minority norm, majority norm) was randomized. Note that the social norm values (37% vs. 74%) for the two experimental conditions follow those selected by Glynn et al. (2009) and Stok et al. (2012). The use of the phrase “MTURKers like yourself” was included to enhance the group reference (cf. Terry and Hogg, 1996). Referencing the behavior of members of this online community was expected to increase potential adherence to social norms as identification with the norm referent group is important for compliance (Turner, 1991).

### Participant Recruitment and Characteristics

Data collection was via the Mechanical Turk platform. This platform was chosen for three reasons: Firstly, all users of the platform are regular users of the Internet. Secondly, these participants are predominantly driven by financial gain and fast task completion, a situation encountered by most individuals working online. Such users will be driven by their primary goal (task completion), which presents an ideal opportunity to explore the choice architecture around privacy and security nudges. Thirdly, using participants from the same occupational group allowed us to make sure that the participants would identify with the referent group mentioned in the social manipulations, in this case MTurkers.

In order to ensure a reasonable sample size to conduct inferential statistics (chi-square and ANCOVA) for the three hypotheses (VanVoorhis and Morgan, 2007), we recruited 309 participants for the study. A small group of cases (*n* = 19) were excluded due to duplication, errors in the data or missing data. The final dataset included 290 cases. Participants were aged on average 35 years (*M* = 35.30, *SD* = 11.96) with an age range between 18 to 71 years old. Just over half (53.1%, *n* = 154) of the participants were women and (45.9%, *n* = 133) male (three missing values).

### Procedure

Before commencement of this study, full ethical approval was received from the Faculty of Health and Life Sciences Ethics Committee at Northumbria University. All participants accessed the study via a link listed on Amazon’s Mechanical Turk platform where they were notified of a flat rate of $1 for participation in the study. Once they had accepted the task and completed the consent form, they were randomly allocated to one of the three cookie conditions. Regardless of their response in the cookie dialog, all participants received the same content thereafter – i.e., they were shown images of four travel websites welcome screen and answered a series of questions about those sites. This was followed by a questionnaire to assess their impulsivity, risk-taking, self-disclosure and sociability. Control questions and demographics were presented toward the end. A debriefing statement followed.

### Personality Measures

Following the incidental task, participants completed several scales designed to measure impulsivity, risk-taking, self-disclosure, sociability, and demographics, with instructions to participants written as follows: “Thank you for completing the task. We would hereby like to ask you a few questions about how you generally make decisions and share information about yourself.” The measures were matched to the constructs of interest discussed in the introduction (risk taking, impulsivity, self-disclosure, and sociability). Note that at various points we used subscales or slightly shortened measures. The advantages to this were (i) a shorter total time on task with the expectation of reduced dropout due to survey length (Hoerger, 2010) and (ii) improved task relevance. Shorter scales featuring three or more items often perform similarly well compared to longer scales when the items feature more than three response options (Peterson, 1994). In each case, the focus was on using well established measures that are readily accessible to enable other researchers to replicate the work. Further, we utilized measures that did not require extensive rewording to be used in an online setting. When shortening the scales, we retained those items that were readily applicable to the online context and our study. All alpha coefficients are given below.

#### Impulsivity

This was measured using five items from the impulsiveness scale by Eysenck et al. (1985). This scale specifically separates impulsivity from risk taking. Instructions asked participants: “Please tell us how you tend to go about making decisions on a day-to-day basis.” The focus was to retain appropriate item stems. Scales with dichotomous response options and fewer number of items tend to have much lower reliability (Peter, 1979). We changed the original questions to personal statements and used Likert-type response options instead of the original dichotomous response options. An example item is: “I buy things on impulse.” Each item included five response options that captured the frequency with which participants engaged in the behaviors, ranging from (1) “never” to (5) “always.” The fourth item was reverse-scored while the fifth item was excluded (α = 0.74, *M* = 2.27, *SD* = 0.58).

#### Risk-Taking

This was measured using three items from Dahlbäck (1990). The scale was taken because it considers risky behavior in relation to other people’s behavior (which was relevant to a study on social influence). An example item is “I think that I am often less cautious than people in general.” The answering options ranged from (1) “strongly disagree” to (5) “strongly agree.” The third item was reverse-scored (α = 0.62, *M* = 2.31, *SD* = 0.73).

#### Self-Disclosure

Self-disclosure was measured with four items from the International Personality Item Pool (IPIP, 2014) and one item by Wheeless (1978). The instructions were as follows: “Please tell us to what extent you share personal information about yourself on a day-to-day basis.” An example item is: “I share and express my private thoughts to others.” The answering options ranged from (1) “never” to (5) “always” (α = 0.77, *M* = 2.93, *SD* = 0.60).

#### Sociability

This was measured using four items from Aluja et al. (2010), retaining the original instructions. An example item is: “I have a rich social life.” The response options ranged from (1) “strongly disagree” to (4) “agree strongly” (α = 0.85, *M* = 2.47, *SD* = 0.73).

#### Control Question and Demographics

Control questions incorporated the following: “Do you normally accept cookies on websites?” Participants could select either “Yes” or “No.” Demographics were also collected, such as age (including an option “prefer not to say”) and gender (including an option “prefer not to say”) in order to describe the sample in later analyses.

## Findings

To explore the hypotheses, we first conducted separate analyses for each hypothesis and then collectively modeled the influence of social framing and personality using logistic regression.

### Cookie Acceptance and Social Framing

As predicted, the use of social norms in the cookie dialog had an effect upon cookie acceptance (see **Table 1**). It was hypothesized that people would reject the cookie if they believed that like-minded others were doing the same. Our findings support this prediction. A Chi-square analysis revealed a statistically significant effect across the three conditions [χ^2^(2) = 22.15, *p* < 0.001] and this finding is supported by Cramer’s V statistics result (Cramer’s *V* = 0.276) that show a moderately strong relationship between social framing and subsequent acceptance (range: 0.25 -0.30). Looking at the cookie acceptance data shown in **Table 1**, we see fewer people accepted the cookie when a minority norm was present– a significant effect as measured by a pairwise comparison [χ^2^(1) = 13.87, *p* < 0.001; φ = -0.266]. Note, however, that there was no statistical difference between the control group and the majority accept group [χ^2^(1) = 0.05, *p* = 0.819]. This is unproblematic, as default settings typically urge participants to accept cookies and so the control condition would simply reflect this bias toward cookie acceptance. For completeness, the two social framing groups also differed significantly from each other [χ^2^(1) = 16.19, *p* < 0.001; φ = 0.283].

**Table 1:** Cookie responses based on social group references (percentages reflect distribution of participants by condition).

	Social framing conditions			
Cookie	Control	Minority (37%)	Majority (74%)	*n*
No (rejected)	22% (19)	47% (51)	20% (19)	89
Yes (accepted)	78% (69)	53% (57)	80% (75)	201
Total	88	108	94	290

### Cookie Acceptance and Personality Effects

We predicted that four personality variables would affect cookie acceptance: impulsivity; risk-taking; self-disclosure and sociability (see means in **Table 2**). To explore this, we divided participants into two groups, those who accepted and those who declined the cookie, and compared each of these traits. We used an analysis of covariance (controlling for age and gender differences in the sample; *p*-values in these analyses were adjusted for multiple comparisons). As predicted participants in the cookie acceptance group had higher impulsivity scores [*F*(1,288) = 6.35, *p* = 0.012, $\eta_{p}^{2}$ = 0.02] and higher risk-taking [*F*(1,288) = 11.66, *p* = 0.001, $\eta_{p}^{2}$ = 0.04] than those in the cookie rejection group. The obtained effect sizes for these results suggest a small to moderate effect of personality. No significant findings were obtained in relation to either willingness to disclose information [*F*(1,288) = 0.03, *p* = 0.857] or sociability [*F*(1,288) = 0.32, *p* = 0.571].

**Table 2:** Means and standard deviations for personality differences by cookie acceptance.

Cookie	Impulsiveness	Risk taking	Sociability	Willingness to disclose information
No (rejected)	2.13 (0.51)	2.09 (0.69)	2.43 (0.72)	2.94 (0.61)
Yes (accepted)	2.32 (0.59)	2.41 (0.73)	2.48 (0.74)	2.93 (0.60)

### Social Framing and Personality

In this section we explore whether those with different personality characteristics might respond differently to the experimental manipulations in terms of their cookie acceptance. As cookie acceptance was associated with greater impulsivity and risk taking, we were interested in exploring potential group differences in relation to these two factors. To achieve this, a median split was performed to create both a high and low impulsive group (median = 2.25) and risk taking group (median = 2.33) (**Table 3**).

**Table 3:** Cookie responses based on social group references and impulsive and risk taking grouping.

	Control		Minority frame		Majority frame	
Cookie acceptance	No	Yes	No	Yes	No	Yes
Low impulsivity	31% (14)	69% (31)	54% (37)	46% (32)	23% (13)	77% (43)
*N*	45		69		56	
High impulsivity	12% (5)	88% (38)	36% (14)	64% (25)	16% (6)	84% (32)
*N*	43		39		38	
Low risk	30% (11)	70% (26)	59% (31)	41% (22)	23% (10)	77% (34)
*n*	37		53		44	
High risk	16% (8)	84% (43)	36% (20)	64% (35)	18% (9)	82% (41)
*n*	51		55		50	

Looking firstly at the high vs. low impulsive individuals depicted in column 1 (control condition), we see that the impulsivity effect was reliable in the control condition – with high impulsive individuals being much more likely to accept the cookie than less impulsive individuals [χ^2^(1) = 4.930, *p* < 0.05; φ = 0.237). However, there was no statistically reliable effect of impulsivity in either the minority [χ^2^(1) = 3.141, *p* = 0.058; φ = 0.171] nor in the majority social framing condition [χ^2^(1) = 0.774, *p* = 0.271; φ = 0.091].

Looking secondly at the high vs. low risk takers, there is a slightly different pattern. There was no significant difference in cookie acceptance between high and low risk takers in either the control [χ^2^(1) = 2.498, *p* = 0.094; φ = 0.168] or the majority framing condition [χ^2^(1) = 0.324, *p* = 0.377; φ = 0.059]. However, there was a significant difference between high and low risk takers in the minority social framing condition [χ^2^(1) = 5.302, *p* < 0.05; φ = 0.222] with 64% of high risk takers accepting the cookie compared to 41% of low risk takers.

These findings suggest that behavioral nudges serve to attenuate individual differences in privacy decision-making that derive from two personality characteristics: impulsivity and risk-taking, although clearly more research would be required to understand the factors at play here.

### Further Analyses

To further investigate these findings, we carried out a hierarchical logistical regression. Hierarchical logistic regression was performed using the Enter method in which model 1 included the control variables (age and gender), model 2 explored the main effect of the social frame, model 3 explored the effect of personality and finally, model 4 explored interactions between the personality variables and social frames. See **Table 4** for model coefficients in predicting cookie acceptance.

**Table 4:** Coefficients of the model predicting whether a user would accept the cookie.

		95% CI for the odds ratio		
Variable	B(SE)	Lower	Odds ratio	Upper
**Model 1**				
Constant	1.106 (0.408)			
Age	-0.010 (0.011)	0.970	0.990	1.011
Gender	0.110 (0.259)	0.672	1.116	1.854
**Model 2**				
Constant	1.569 (0.481)			
Age	-0.008 (0.011)	0.970	0.992	1.014
Gender	0.018 (0.270)	0.599	1.018	1.729
Social frame (control)	–	–	–	-
Social frame (minority)	-1.148 (0.326)^∗∗^	0.168	0.317	.600
Social frame (majority)	0.064 (0.366)	0.520	1.066	2.185
**Model 3**				
Constant	-0.092 (1.076)		
Age	-0.002 (0.012)			
Gender	0.214 (0.291)	0.701	1.238	2.189
Social frame (control)	–	–	–	-
Social frame (minority)	-1.112 (.333)^∗∗^	0.171	0.329	.631
Social frame (majority)	0.114 (0.375)	0.537	1.120	2.337
Risk taking	0.633 (0.262)^∗^	1.127	1.883	3.148
Self-disclosure	-0.163 (.230)	0.541	0.850	1.334
Sociability	0.063 (0.194)	0.728	1.065	1.558
Impulsivity	0.101 (0.310)	0.603	1.106	2.030

*^∗^p < 0.01, ^∗∗^p < 0.05.*

The results of first model, including the control variables (age and gender), revealed a poor fit to the data [χ^2^(2) = 0.94, *p* = 0.625]. After controlling for age and gender, the social framing in Model 2 was found to significantly improve the model [χ^2^(4) = 20.00, *p* < 0.05] explaining between 6 and 10% of the variance in cookie acceptance (Cox and Snell *R^2^* = 0.07; Nagelkerke *R^2^* = 0.10, Hosmer and Lemeshow, *R^2^* = 0.06). Overall, 69.3% of cases were correctly classified in this model and the Wald statistic result revealed that only social frame (minority) contributed significantly to the prediction of cookie acceptance (Wald = 12.44, *b* = -1.15, df = 1, *p* < 0.001). This was due to the fact that the control group was used as the referent group in the model (our earlier findings demonstrated that the control and majority referent group accepted cookies in a similar pattern). The minority framing has a significant negative co-efficient indicating that the presence of a minority social referent is less likely to lead to cookie acceptance. The odds ratio is less than 1, indicating that as the predictor increases (or allocation to this group) the odds of the outcome occurring decrease. Conversely, the majority social referent odds ratio is 1.07 indicating that people in the majority frame are 1.07 times more likely to accept the cookie. The majority social referent does not significantly predict cookie acceptance.

Model 3 introduced the personality factors which further improved the model fit [χ^2^(4) = 12.09, *p* < 0.05] explaining between 9 and 15% of the variance in cookie acceptance (Cox and Snell *R^2^* = 0.11; Nagelkerke *R^2^* = 0.15, Hosmer and Lemeshow, *R^2^* = 0.09) and raised the classification of cases to 71.4%. The *Wald* statistic result revealed that only one personality factor contributed significantly to the prediction of cookie acceptance, which was risk taking (Wald = 5.84, *b* = 0.63, df = 1, *p* < 0.05).

Model 4 did not lead to a significant change in the model [χ^2^(14) = 10.73, *p* = 0.707] indicating that there were no interactions between social framing and personality, and interactions between personality factors. Model 3 was therefore the best fit to the sampled data. Risk taking has a significant positive co-efficient and an odds ratio of 1.88, indicating that individuals higher in risk taking are 1.88 times more likely to accept the cookie.

The results of logistic regression suggested a slightly different picture compared to our reported analysis. The regression outcomes confirm our chi-square findings regarding differences in social framing. However, only risk-taking contributes significantly to the prediction of cookie acceptance in the regression model. The non-significant result for impulsivity is likely due to two aspects: (1) variable reduction propensity of logistic regression and (2) a potential suppression effect [the correlation between impulsivity and risk taking is significant (*r* = 0.589, *p* < 0.001)]. Using chi-squared analyses overcomes these limitations and isolates the influence of these two factors. Impulsivity significantly predicts acceptance when risk-taking is removed demonstrating the high inter-correlation between the two. Lastly, we are unable to identify interactions between personality types and the social framing. The large number of predictors in the logistic regression and the inter-correlations means that these potential interactions did not appear as the regression is confounded.

## Discussion

In this study we measured cookie acceptance directly rather than as a self-reported behavior. This is important as avowed intentions do not translate well into actual behaviors – a major concern for human-centric studies in privacy and security according to Crossler et al. (2013). We explored social framing effects by invoking different social norms in a cookie dialog and found that those norms that indicated low cookie acceptance were able to drive people away from the ‘default’ position of accepting the cookie. We also explored the personality characteristics of risk-taking, impulsivity, sociability and self-disclosure, noting an influence of the first two on cookie acceptance. However, risk taking was found to be the more reliable predictor. We did not find evidence for an interaction between the personality factors and the social norm conditions. We capture our predictions and interpretation of findings in more detail in the next section, before moving on to a discussion of the limitations and implications of the work and conclusion.

### Interpretation of Findings

The first hypothesis predicted that a social nudge might be capable of moving people away from accepting cookies as the normative or ‘control behavior.’ This hypothesis was supported. When a cookie dialog included a reference to low cookie acceptance rate as a social norm, participants were nudged away from accepting the cookie. These results are in line with previous findings that illustrate behavior change in the face of social nudges (Stok et al., 2012) and those studies showing the effects of social nudges on privacy behavior (Goecks and Mynatt, 2005; Besmer et al., 2010). Note that the presence of a majority social acceptance norm in the cookie message did not generate any systematic change in behavior, as compared to a control condition – presumably because the ‘majority norm’ was more reflective of default acceptance behaviors. We note the implications here for the current and future European Union directives around the use of cookies – with current recognition of cookie acceptance as the default, but the possible introduction of more detailed information about the privacy implications of cookies to come. Our research thus contributes to current understanding in respect of ‘social proof’, building on the work of Das et al. (2014) and recognizing evidence from other contexts (see Glynn et al., 2009; Stok et al., 2012). Taken together, these show how perceived social norms can directly affect decision-making.

The second hypothesis focused on the role of personality in decision-making, following work by Riquelme and Román (2014). It was hypothesized that participants would be more likely to accept cookies if they were also (i) more impulsive, (ii) more willing to share information, (iii) greater risk takers and (iv) more sociable. This hypothesis was supported for only two of the four traits (impulsivity and risk-taking), but most strongly for risk taking. There is a suggestion that more impulsive individuals were more likely to accept cookies, which also fits with findings that they are less likely to deliberate on their options (see Halpern, 2002; Magid and Colder, 2007). Risk taking was a stronger predictor of cookie acceptance than impulsivity in this study. Risk taking has been found to be a predictor of other security behaviors (see Grossklags and Reitter, 2014). This supports findings by Zeelenberg et al. (1996) that risk takers are less likely to be concerned about the repercussions of their actions and less likely to consider any subsequent regret.

No evidence was found to link cookie acceptance to either self-disclosure or sociability. Perhaps the association between cookie acceptance and self-disclosure as well as privacy concern was too tenuous. Participants may not have recognized that cookies may also involve sharing potentially identifying and/or personal information. This suggests that context-dependency plays an important role, as different environmental cues may influence disclosure of private information (John et al., 2011).

Nurse et al. (2011) proposed that it is imperative to design systems and communication in a way that caters to individual strengths. Personality traits may shape user strengths and weaknesses in relation to privacy and security behaviors. Based on our results, in the absence of a nudge, risk taking and impulsivity may represent potentially unfavorable traits in security-related decision making. Our third hypothesis focused on the intersection between personality differences and social framing. The study explored this in relation to levels of impulsivity and risk-taking by investigating group differences. We do not wish to put too much weight on these findings, as our analysis relied on a median split between high vs. low impulsive and high vs. low risk-takers that compared groups of uneven sizes; however, we would draw attention to the fact that nudges were effective for vulnerable cohorts such as high risk-takers. This is important when considering privacy-by-design approaches that rely upon an understanding of human behavior, as we rarely consider the ways in which behavioral nudges might be targeted at specific personality types. Thus, for example, nudges could be employed to move risk-taking individuals away from problematic default behaviors. It is easy to understand how such a social manipulation might act to improve the privacy and security behaviors of risk-takers without unnecessarily triggering the concerns of those who are already risk-averse.

Our study does have its limitations. In measuring cookie acceptance behavior directly, we were reduced to reporting a binary cookie acceptance decision that then limited our ability to perform a meaningful parametric assessment of interaction effects in respect of personality and social framing. Also, while we found that social nudging was effective, our interventions may have produced only short-term behavioral change and we must recognize that other forms of enquiry would be needed to explore longer term, enduring behavior change and norm internalization (e.g., Mols et al., 2015). Finally, the current study took place in a research environment where security may not have been perceived as a matter of concern. MTurkers may have assumed that the study would not pose any danger to them, reducing their concerns about potential security risks. This may not be representative of other groups or contexts and may affect the generalizability of the presented findings beyond MTurk (Casler et al., 2013; Paolacci and Chandler, 2014).

### Practical Recommendations and Future Research

To date, a number of researchers have exploited known cognitive biases and habits in order to help users make more informed choices (Acquisti, 2009). For example, privacy nudges have been employed to reduce an unintended disclosure of information to a wider audience on social media sites (Wang et al., 2014), to influence privacy settings on mobile phones and similar devices (Choe et al., 2013; Turland et al., 2015), or to nudge individuals away from privacy-invasive apps (see Choe et al., 2013). In a related security domain, nudges have also been used to reduce the “update later” response to security updates (Zhang-Kennedy et al., 2014); to create more secure passwords (Forget et al., 2008). We have added to this work, but note the following recommendations for future work. Firstly, nudges would appear to be most effective when they are present at the point that a decision is to be made, but we know relatively little about the way their influence may wane over time. Secondly social norm nudges ought to be credible and care must be taken in regard to the way that they map onto existing social norms (e.g., Stok et al., 2012). In our own data we saw little evidence for the influence of a ‘majority norm’ and have interpreted this in terms of existing default behaviors. However, it is interesting to note, in the data presented in **Table 3**, the possibility of a personality linked baseline effect in which those more conservative individuals with a relatively low cookie acceptance rate might be influenced to accept cookies when presented with a majority social norm. Baseline behaviors are important when exploring norm-based nudges – something that has been observed in relation to the use of social nudges in other contexts (Allcott, 2011). Norm-based nudges are therefore likely to be maximally useful when the default behavior is well known.

A variety of other future research avenues are also worth considering. First, work on affect and risk communication (Visschers et al., 2012) may provide further ideas for nudging in the area of information security. Second, the result of framing in tasks may also be influenced by other individual characteristics not under investigation in this study (Tversky and Kahneman, 1981; Kühberger, 1998), including conscientiousness, agreeableness (Levin et al., 2002), risk preferences and gender (Druckman, 2001; Levin et al., 2002). More research on framing effects and the role of individual differences on decision-making (see Levin et al., 2002) may help us to better understand under what circumstances and for whom framing is effective. This would then allow practitioners to develop choice architectures capable of exerting the greatest influence. We note, here, that behavioral researchers working in other contexts have shown the importance of individual differences in the appropriate tailoring of a behavioral nudge. Thus, for example, Costa and Kahn (2013), working in the field of energy conservation, found that the provision of a report on self and peer consumption of energy was an effective nudge for political liberals but was ineffective with political conservatives. Finally, it is likely that the effectiveness of social norms may be dependent on the extent to which the individual identifies with the referent group (Stok et al., 2012). Future research may wish to explore this in more depth as part of a wider remit to understand the way that tailored social information may influence an individual.

## Author Contributions

The authors worked as a team and made contributions throughout. PB and LC conceived of the research program as part of the original funding bid and led the team, contributing to design, the interpretation of data and the final stages of writing up. DJ as lead researcher, contributed to the design, supervised the running of the project, conducted the statistical analyses together with JB and wrote the first draft of the manuscript. JT was responsible for designing and building the data collection platform.

## Conflict of Interest Statement

The authors declare that the research was conducted in the absence of any commercial or financial relationships that could be construed as a potential conflict of interest.
